# The role of energy deposition on the luminescence sensitization in porphyrin-functionalized SiO_2_/ZnO nanoparticles under X-ray excitation†

**DOI:** 10.1039/d4na00640b

**Published:** 2025-01-08

**Authors:** Irene Villa, Roberta Crapanzano, Silvia Mostoni, Anne-Laure Bulin, Massimiliano D'Arienzo, Barbara Di Credico, Anna Vedda, Roberto Scotti, Mauro Fasoli

**Affiliations:** a Department of Materials Science, University of Milano-Bicocca Via Cozzi 55 Milano I-20125 Italy irene.villa@unimib.it; b INSTM, University of Milano-Bicocca Via Cozzi 55 Milano I-20125 Italy; c University Grenoble Alpes, Inserm U1209, CNRS UMR5309, Cancer Targets and Experimental Therapeutics Team, Institute for Advanced Biosciences Grenoble 38000 France; d Institute for Photonics and Nanotechnologies-CNR Via alla Cascata 56/C Povo (TN) 38123 Italy

## Abstract

Hybrid nanoscintillators, which feature a heavy inorganic nanoparticle conjugated with an organic emitter, represent a promising avenue for advancements in diverse fields, including high-energy physics, homeland security, and biomedicine. Many research studies have shown the suitability of hybrid nanoscintillators for radiation oncology, showing potential to improve therapeutic results compared to traditional protocols. In this work, we studied SiO_2_/ZnO nanoparticles functionalized with porphyrin as a photosensitizer, capable of producing cancer cytotoxic reactive oxygen species for possible use in radio-oncological therapeutics. Radioluminescence measurements under increasing energy of the ionizing radiation beam up to 10 keV show sensitization of porphyrin moieties on SiO_2_/ZnO. This can be attributed to an increase in energy deposition promoted by the ZnO nanoparticles, which have a higher density and atomic number. This assumption was confirmed by computational simulations of energy deposition after the first interaction of ionizing radiation with SiO_2_, ZnO, and air. Indeed, Monte Carlo simulations evidence that, despite a decrease in the absolute number of X-rays interacting within the system while increasing the energy of the beam, at 10 keV, the presence of ZnO is dominant to enhance energy deposition. Hence, these experimental and computational studies evidence the importance of each hybrid nanosystem component in the scintillation process. This work shows how an appropriate choice of constituents, in terms of physicochemical properties and architecture, can favour energy deposition mechanisms under X-ray irradiation and thus can boost the hybrid nanosystems' performance for diverse biomedical scintillation-based applications.

## Abstract 2

Upon X-ray irradiation, the dense ZnO-related enhancement of energy deposition in TCPP-functionalized SiO_2_/ZnO nanoparticles is the mechanism that cooperates to activate the sensitization of TCPP luminescence (RL).

## Graphical abstract

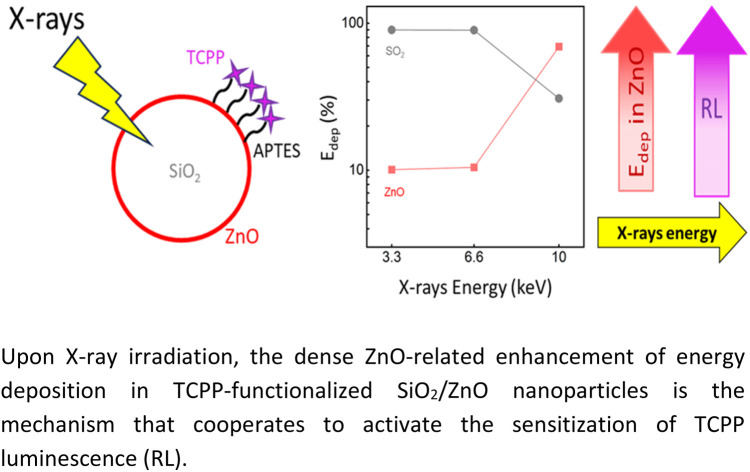

## 1 Introduction

Scintillating nanoparticles (NPs), or nanoscintillators (NS), are widely exploited in advanced detection technologies, such as high-energy physics, border control security, radioactive gas detection, and medical diagnostics.^1–5^ Moreover, dense inorganic NPs, such as metals (*e.g.* Au NPs) and metal oxides (*e.g.* TiO_2_, HfO_2_, or ZnO NPs), act as radiosensitizers and radiocatalysts, and they have been proposed as co-adjuvant of the traditional radiotherapy (RT) for cancer and other disease treatments. These systems aim to increase the therapeutic outcomes, reduce mortality and recurrence of the illness, and improve patients' quality of life.^6–11^ Recently, a new concept of hybrid NS has gained attention. They are composed of inorganic scintillating NPs coupled with organic dye molecules. The inorganic nanoparticles feature high density and a high effective atomic number (*Z*_eff_), which favour interactions with ionizing radiation, whereas the organic molecules can exhibit distinct qualities according to the targeted applications. For example, when hybrid nanosystems are proposed for X-ray-activated photodynamic therapy (X-PDT) for the treatment of deep-seated cancer and other mortal diseases, the organic moiety grafted on the surface of NPs for X-PDT is typically a photosensitizer (PS), such as porphyrin molecules, able to produce cytotoxic reactive oxygen species (ROS).^12–16^ Due to an appreciable spectrum overlap between the scintillator's radioluminescence emission and the photosensitizer's absorption band, and short-range packing of the NS and the PS, the PS activation is expected to be triggered both by radiative and/or non-radiative energy transfer (ET) mechanisms and by energy deposition within the system.^17–19^ A plethora of dense NS has come to the attention of the medical and scientific communities for X-PDT purposes. Quantum dots, metal/rare earth oxides, fluorides, and semiconductors have been studied in this context to excite conjugated photosensitizers and produce cytotoxic singlet oxygen.^20–22^ Diverse doping strategies on NS have been shown to improve the emission of NS with beneficial therapeutic consequences.^23^ This is the case of fluorides and oxides scintillators^24–28^ – such as NaLuF_4_:Tb^3+^, NaGdF_4_:Eu^3+^, LaF_3_:Ce^3+^,Tb^3+^, Gd_2_O_3_:Eu^3+^, and SrAl_2_O_4_:Eu^2+^ – where the augmented dopant ions' emission – lying typically in the visible spectral range – matches the absorbance of the majority of organic photosensitizers (*e.g.*, Rose Bengal, porphyrin, and protoporphyrin IX) for proper activation of their cytotoxicity.^29^

Although many authors have demonstrated the great outcomes of X-PDT deriving from the exploitation of dense hybrid nanosystems, the interplay among the diverse mechanisms likely to excite the PS under ionizing radiation remains a matter of debate. In fact, for hybrid NS-triggered X-PDT, ionizing radiations may interact either with the high *Z*_eff_ NPs or with the biological surroundings. When interacting with high *Z*_eff_ NPs, secondary energetic ionized charges are created with travel distances that exceed the nanometric scale. Thus, parts of these ionized charges escape from the NP. How the charges deposit energy within the NPs and the surrounding biological medium is crucial since it can affect the therapeutic dose and the ROS generation by activating nearby PS. It is worth mentioning that the fraction of energy deposited in the NPs varies with their density, *Z*_eff_ and their size, together with the energy of the incoming beams.^18,22,30–32^ The complexity of this picture denotes the urgency of an accurate investigation of the energy deposition and scintillation mechanisms able to activate organic molecules in hybrid nanosystems towards a more efficient RT and X-PDT. This work focuses on a model hybrid NS made of scintillating high-density ZnO NPs deposited on a SiO_2_ support (SiO_2_/ZnO), functionalized with metal-free porphyrin as conventional PS through established SiO_2_ surface functionalization approaches.^33–37^ It is worth mentioning that the results obtained in this work on the luminescence properties of bare and porphyrin-functionalized SiO_2_/ZnO systems are supported and compared to the ones previously described.^38,39^ However, besides the investigation of the spectroscopic properties, this work also provides a fresh model of the energy deposition mechanisms occurring in the dense core of the hybrid SiO_2_/ZnO nanosystems. Zinc oxide is an excellent photocatalyst for photodegradation of the highly toxic pollutants in aqueous environments.^40^ Lately, biocompatible ZnO and ZnO-based systems have been promoted for biomedical applications^41–46^ and for radiocatalytic activities inducing radiosensitization in RT.^47^ In this framework, ZnO NP presents an intrinsic greenish emission under optical and X-ray irradiation^48,49^ that is resonant with the porphyrin absorption, and, thus, it is exploitable for the activation of the X-PDT mechanism. For these reasons, SiO_2_/ZnO was selected as a model material for this study. We provide a deeper experimental investigation of the RL characteristics of such systems under X-ray irradiations as a function of the energy of the incident beam in the range accessible by our experimental setup. The X-ray energies are in the keV region and the spectroscopic results are related to the compositional features of the systems. The Geant4-based computational modelling is used to estimate the spatial distribution profile of the deposited energy in SiO_2_/ZnO at increasing energy of the X-rays beam. The combination of experimental techniques and theoretical simulations helps to comprehend the mechanisms of interactions between ionizing radiations and hybrid nanoscintillator. In particular, our findings evidence the critical importance of the presence of dense components, such as ZnO, in the energy deposition process at increasing energies of the X-ray radiation. The consequent increment of energy released can be the key tool to boost the therapeutic activity of NS-based radiation oncology protocols performed with high-energy X-ray beams.

## 2 Methods

### 2.1 Synthesis of SiO_2_/ZnO nanosystems and functionalization by porphyrin moieties

Precursors for the synthesis of SiO_2_/ZnO NS were purchased from Merck Life Science and Exacta Optech Labcenter. For the functionalization, 5,10,15,20-(tetra-4-carboxyphenyl) porphyrin (TCPP, 98%) was purchased from PorphyChem SAS; (3-aminopropyl)triethoxysilane (H_2_N(CH_2_)3Si(OC_2_H_5_),^3^ APTES, 98%), *N*,*N*-dimethylformamide (DMF, 99%) and toluene (99%) from Alfa Aesar.

First, spherical SiO_2_ NPs were synthesized according to ref. 50 by quickly adding tetraethylorthosilicate (TEOS, 0.28 M) to an ethanol solution with water (6 M) and NH_4_OH (0.40 M). Then, ZnO NPs were grown onto the surface of the so-prepared SiO_2_ NPs by the hydrolysis and condensation of Zn(CH_3_COO)*2H_2_O in a basic ethanol solution.^39,51^ The final ZnO loading was about 6.6 wt%, indicating that, on average, approximately 215 ZnO NPs were anchored to the available surface of each SiO_2_ NP. The functionalization step was carried out as reported in ref. 38. The SiO_2_/ZnO nanosystems were decorated with 0.2 wt% of APTES, used as a surface ligand. This sample was labelled as SiO_2_/ZnO@APTES. Subsequently, two different concentrations of TCPP were covalently coupled to the SiO_2_/ZnO@APTES NPs through the APTES amino groups, corresponding to APTES:TCPP molar ratios of 1 : 0.1 (SiO_2_/ZnO@APTES@Porp 0.1%) and 1 : 1 (SiO_2_/ZnO@APTES@Porp 1%), and to dye loading levels of 0.3 wt% and 2.7 wt%, respectively.

### 2.2 Structural and spectroscopic characterization

#### 2.2.1 Transmission electron microscopy (TEM)

TEM images were collected using a JEOL JEM-2100Plus TEM operating with an acceleration voltage of 200 kV, equipped with an 8-megapixel Gatan RioTM complementary metal-oxide-semiconductor camera. The samples were deposited onto carbon-coated Cu TEM mesh grids by drop-casting dilute NPs dispersions in EtOH. The sizes of SiO_2_ and ZnO NPs were determined from TEM images on a set of hundred NPs.

#### 2.2.2 Thermogravimetric analysis (TGA)

TGA was performed with a Mettler Toledo TGA/DSC1 STARe System (30 °C-1000 °C, heating rate of 10 °C min^−1^, constant air flux of 50 mL min^−1^). The weight loss between 150 °C and 1000 °C (Δ*W*_150 °C–1000 °C_) was used to calculate (i) the amounts of both APTES and TCPP in the functionalized samples (TCPP amount, wt%) and (ii) the ratio between APTES and TCPP molecules distributed over SiO_2_ surface (number of molecules per nm^2^ SiO_2_) according to ref. 38 The uncertainty on the quantitative determination was evaluated as a mean of three different TGA analysis run on each sample.

#### 2.2.3 UV-vis absorption measurements

UV-vis experiments were conducted to quantify the TCPP amount in SiO_2_/ZnO@APTES@Porp 0.1% and SiO_2_/ZnO@APTES@Porp 1%. The UV-vis experiments were performed with an Agilent Cary 100 spectrophotometer in the spectral range of 300–700 nm. Spectra were recorded in quartz cuvettes with 0.1 cm optical path length. Double measurements were recorded for each sample. The absorbance of TCPP was measured in highly diluted powder dispersions (1.3 mg powder/mL in DMF, corresponding to 10^−3^ M of ZnO). The data were obtained by using the Lambert–Beer equation on the absorbance value of TCPP at 520 nm, corresponding to the first TCPP Q-band (molar extinction coefficient at 520 nm *ε*__520_ = 8900 M^−1^ cm^−1^, optical path length *d* = 0.1 cm) as in ref. 38 (see Table S2 in the ESI†).

#### 2.2.4 Radioluminescence (RL) under soft X-ray excitation at diverse energy

Steady state radioluminescence (RL) measurements were carried out at room temperature using a homemade apparatus featuring, as a detection system, a liquid nitrogen-cooled, back-illuminated, and UV-enhanced charge-coupled device (CCD) Jobin-Yvon Symphony II, combined with a monochromator Jobin-Yvon Triax 180 equipped with a 100 lines per mm grating. All spectra were corrected for the spectral response of the detection system, after the subtraction of the background. RL excitation was obtained from the unfiltered X-ray irradiation through a Be window, using a Philips 2274 X-ray tube with a tungsten target operated at 10 kV, 20 kV, and 30 kV. The corresponding mean energy of the generated X-ray beam was 3.3 keV, 6.6 keV, and 10 keV.^52^

For consistency and reproducibility of the RL experiments, X-ray irradiation was performed only on powder samples. The powders of bare SiO_2_/ZnO, TCPP-functionalized (at concentrations of 0.1% and 1%) SiO_2_/ZnO and the pure TCPP porphyrin photosensitizer were placed in an aluminium crucible with dimensions of 8.5 mm diameter and 1 mm height.

### 2.3 Computational details

The energy deposition spatial profile in SiO_2_/ZnO nanosystems was computationally evaluated by Monte Carlo simulations using the GEANT4 toolkit (version 4.10.6 patch 01) through the developments of two codes (codes A and B) simulating two diverse assemblies of SiO_2_/ZnO in air. The computational analyses were performed on systems mimicking the morphology of real samples and with energy parameters simulating the experimental RL conditions whenever the computational capacity allowed it. In other cases, appropriate approximations were considered. First, the Low-Energy Electromagnetic Physics-Livermore package with a cutting step of 1 nm was used since it permits the computation of the interactions of electrons and photons with inorganic materials down to approximately 250 eV. Then, the X-ray irradiation was modelled as a beam of 1 × 10^8^ parallel photons, generated at a distance of 15 cm with a tilt angle of 26° with respect to the SiO_2_/ZnO nanosystem. The monochromatic energies of the X-ray beam correspond to the average ones used in the experimental RL measurements, *i.e.* 3.3 keV, 6.6 keV, and 10 keV.

#### 2.3.1 Code A

A single sphere of SiO_2_ with a diameter of 80 nm was placed in an air environment and covered by an increasing number (0, 10, 100, and 215) of spherical ZnO NPs of 5 nm diameter. The ZnO NPs were randomly placed on the surface of SiO_2_ without any overlap. The energy deposition was then recorded according to where the first interaction between X-ray and the nanosystem occurred: the energy spatial profile was represented as a function of *r*_SiO_2__ and *r*_ZnO_ when the first interaction occurred in SiO_2_ or ZnO, respectively.

#### 2.3.2 Code B

To faithfully represent the system used for the RL experiments, a cylindric sample holder (8.5 mm diameter, 1 mm height) containing air should be filled by randomly placing 3.9 × 10^16^ SiO_2_ spheres (80 nm diameter) each covered by 215 spherical NPs of ZnO (5 nm diameter). This configuration largely exceeded the computational capabilities. Therefore, three main approximations were made: (i) the dimensions of the sample holder were downscaled to 8.5 μm diameter and 1 μm height, maintaining the same size ratio, (ii) accordingly, the number of NPs was decreased to 5 × 10^4^ keeping a mean inter-distance of ∼50 nm between them, and (iii) for each SiO_2_, the 215 ZnO NPs were replaced by a spherical shell of 0.7 nm thickness of ZnO placed around the silica surface. The thickness of the shell was calculated in the way that its volume was equal to the one occupied by 215 NPs.

## 3 Results and discussion

### 3.1 Morphological and radioluminescence properties of porphyrins functionalized NPs

**Fig. 1:**
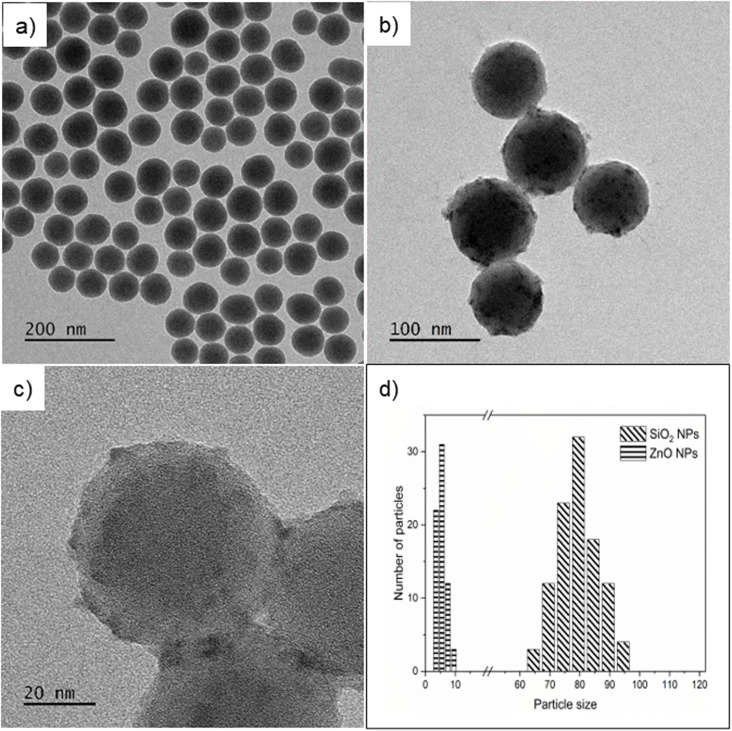
(a) TEM image of pristine Stöber silica NPs; (b and c) TEM image of SiO_2_/ZnO and SiO_2_/ZnO@APTES@Porp 1% NPs; and (d) particle-size distribution evaluated by measuring the diameter of ∼150 SiO_2_ and ∼80 ZnO NPs in SiO_2_/ZnO@APTES@Porp 1% sample.

The structural and morphological analysis of bare and porphyrin-functionalized SiO_2_/ZnO nanosystems has been previously detailed.^38,39^ However, some critical morphological details are reported here to discuss their relationship with the scintillation process and the energy deposition mechanisms occurring in the systems under X-ray irradiation with increasing energy. The synthesis of SiO_2_/ZnO@APTES@Porp employs homogeneous and spherical silica Stöber NPs with an average size of 80 ± 5 nm (TEM, Fig. 1a) decorated with ZnO NPs of about 5 nm in diameter (Fig. 1b). Upon the functionalization with APTES and the grafting with TCPP, the morphological features of the sample do not disclose further variations (Fig. 1c and d). TGA thermal profiles were recorded for all samples to quantify the presence of APTES and TCPP molecules after the functionalization step. The weight loss between 150 °C and 1000 °C increases coherently with the amount of APTES and TCPP (Fig. S1 in the ESI†). In addition, the APTES and TCPP amounts, as well as the APTES:TCPP ratios, were estimated from the TGA measurements according to the method reported in ref. 38 (Table S1 in the ESI†). The quantity of TCPP grafted onto SiO_2_/ZnO@APTES@Porp 0.1% and SiO_2_/ZnO@APTES@Porp 1% was also assessed through UV-vis experiments by measuring the absorbance of the Q-band of TCPP at 520 nm. The recorded values are reported in Table S2 in ESI† and appear in agreement with the ones obtained from TGA.

**Fig. 2:**
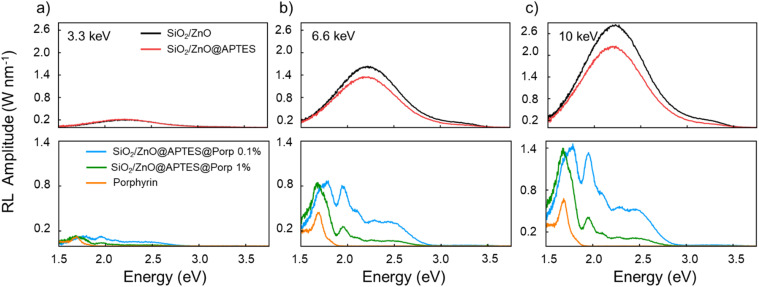
RL spectra as a function of the energy (eV) on powders of the bare (SiO_2_/ZnO and SiO_2_/ZnO@APTES) and porphyrin-functionalized nanosystems (SiO_2_/ZnO@APTES@Porp 0.1% and SiO_2_/ZnO@APTES@Porp 1%) recorded under excitations with X-ray beam of 3.3 (a)/6.6 (b)/10.0 (c) keV mean energy. For comparison, the RL spectra of the powder of porphyrin alone are also represented.

The powders of bare and functionalized SiO_2_/ZnO nanosystems along with the powder of porphyrin moieties were investigated by excitation with X-ray irradiation with increasing average energies (3.3 keV, 6.6 keV, and 10 keV) (Fig. 2) in order to explore the dependence of the RL features with the X-ray energy. All the RL spectra could be fitted in a suitable way by the sum of the same Gaussian components set reported in Table S3.† At least six components were mandatory to count for the ZnO emission in the UV-vis spectral range and for the porphyrin red luminescence. Interestingly, in order to obtain a satisfactory fit of the experimental RL curves of the functionalized SiO_2_/ZnO, four additional Gaussian components, with negative intensity, were required and peaked in the wavelength spectral range from 500 nm to 600 nm, where the porphyrin absorption Q-bands appeared (*vide infra*).^53^ In detail, in Fig. 2a–c (top panels) and S2–S4 (top panels in the ESI†), the RL emission of bare SiO_2_/ZnO nanosystems is reported. In these spectra, a broad visible emission centred at ∼2.22 eV (563 nm) attributed to ZnO defect centres and a weaker excitonic UV band at ∼3.09 eV (400 nm) appeared, regardless of the X-ray energy. The silica substrate shows only a negligible RL signal at ∼4 eV (310 nm) (Fig. S5†), as already reported in ref. 39 on the similarly synthesized NS. The RL of APTES-functionalized NP presents the same spectral components of SiO_2_/ZnO, suggesting that the ligand does not introduce new optically active centres. The RL of TCPP-functionalized nanosystems is composed of the ZnO luminescence bands together with the porphyrin-related bands from 1.5 eV (826 nm) to 2 eV (620 nm) (Fig. 2a–c bottom panels, S2–S4 bottom panels), associated with J aggregates, porphyrin monomers, and complexes formed by the released Zn^2+^ ions and the aromatic central ring of porphyrins.^38,54,55^ It is worth noticing that the relative intensities among the RL bands in the functionalized samples change according to the TCPP loading level. More specifically, in SiO_2_/ZnO@APTES@Porp 1%, the RL emission is dominated by the band at 1.7 eV, which is ascribed to the presence of more J-aggregates species with respect to the sample with lower dye concentration. Consistently, the RL spectra of the porphyrin powder (dye content of 100 wt%) feature only the aggregates emission. The detailed description of the RL behaviour of these nanosystems as a function of the TCPP loading level, the spatial distributions between the SiO_2_/ZnO, and the dye moieties has already been published.^38^ In the numerical fit of the spectra of TCPP-functionalized SiO_2_/ZnO, besides the luminescence bands of ZnO, four Gaussian components with negative areas were added to consider the absorption of the porphyrin Q-bands (Fig. S2–S4†). This allowed us to describe the presence of dips in the RL spectra occurring at 2.01 eV (617 nm), 2.2 eV (564 nm), 2.34 eV (530 nm), and 2.5 eV (496 nm) (Fig. 2, bottom panels). These dips suggest a possible re-absorption of the ZnO luminescence by the TCPP porphyrin molecules. Also, the slight disagreement of the cumulative fit with respect to the experimental data in the range above 3 eV can be due to the re-absorption of the ZnO RL by the tail of the porphyrin's Soret band around 3.1 eV (400 nm) that was not included in the numerical fit. Therefore, a radiative ET mechanism partially allows the activation of the red emission of the PS, as previously reported in ref. 38 for identical systems. In general, the experimental and the numerical fit results highpoint that the RL intensity of all the nanosystems is enhanced by increasing the energy of the X-ray photons. Moreover, Fig. 2 displays that the RL intensity of the porphyrin is weaker in the TCPP alone than in the functionalized NS at any X-ray beam energy, due to the weak probability of interaction with ionizing radiation in the keV energies range related to its low density (TCPP *ρ* = 1.3 g cm^−3^).^56^ On the contrary, the TCPP red luminescence is sensitized during the hybrid NS, especially at 6.6 keV and 10 keV. This can be attributed to the presence of a scintillating core with a density higher than the one of the TCPP itself. Indeed, the ZnO NPs are the heaviest elements of the hybrid nanosystems (*Z*_Zn_ = 30, *Z*_O_ = 8, *Z*_ZnO_ = 27.8,^57^*ρ*_ZnO_ = 5.61 g cm^−3^) thus, they can more efficiently interact with soft X-rays, mainly through the photoelectric effect. After the first interaction in ZnO NPs, the X-ray energy is deposited in the heavy components and the surrounding medium by the produced secondary ionized charges. On the TCPP-functionalized SiO_2_/ZnO surfaces, the organic molecules are in proximity to the dense NPs, which promote the energy deposition strictly close to the dye. This means that, at the higher energies of the X-ray beam, the increment of the RL red luminescence of the porphyrins is expected to be beneficially driven by the energy deposition augmentation due to ZnO. Moreover, whenever the primary interaction of the X-ray beam occurs in SiO_2_, this latter might have a partial role in the energy deposition mechanism thanks to its medium density (*vide infra*) (*Z*_Si_ = 14, *Z*_O_ = 8, *ρ*_SiO2_ = 2.65 g cm^−3^).

Understanding the role of the energy deposition as a function of the energy of the incoming X-rays and the density and dimension of each element composing the sample is tricky. In this work, a complementary computational model was developed to find a rationale for the experimental RL results evidencing diverse efficacies in sensitization of the TCPP according to the energy of the incident beam and to shed light on the energy deposition profile features in hybrid nanosystems under ionizing radiation.

### 3.2 Geant4 simulations and energy deposition profile *vs.* X-ray beam energy

**Fig. 3:**
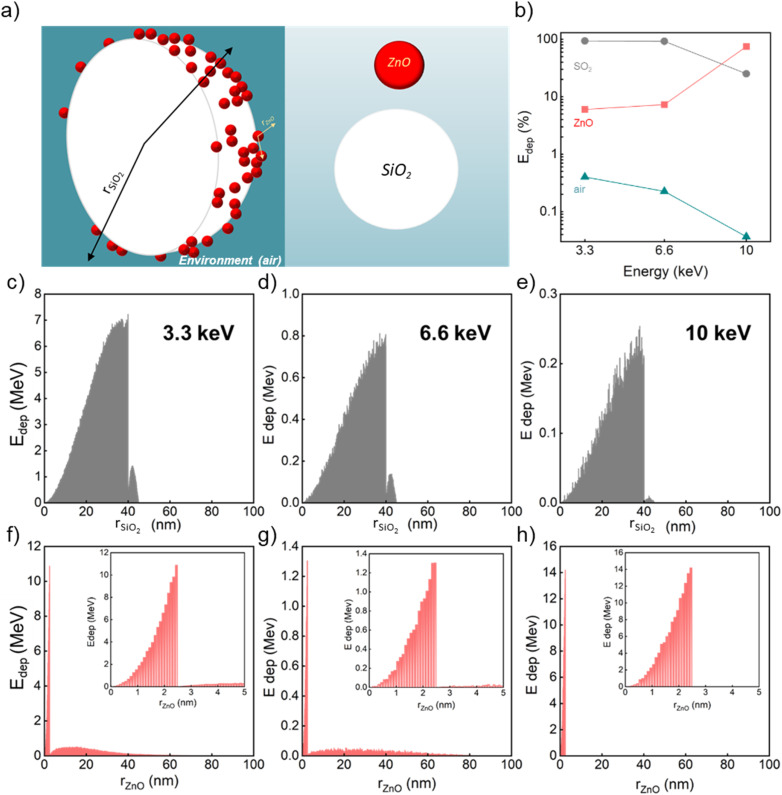
(a) Visualization of the geometry simulated in code A: small spheres of ZnO NPs (red, 5 nm diameter) are randomly distributed on the surface of a SiO_2_ sphere (white, 80 nm diameter) placed in air (teal green). We alternatively simulated 0, 10, 100, and 215 ZnO NPs. The radii of the SiO_2_ and ZnO NPs are depicted (black and pale orange arrows, respectively). (b) Percentage of energy deposited (*E*_dep_ (%)) in ZnO, SiO_2_, and air as a function of the X-ray energy; calculated using code A and reported in Table 1. (c–e) Energy deposited in the SiO_2_/ZnO system plotted as a function of *r*_SiO_2__ when X-rays of various energy interact first with SiO_2_. In these simulations, 215 ZnO NPs were randomly placed on the surface of the SiO_2_ NP. (f–h) Energy deposited in the SiO_2_/ZnO system plotted as a function of *r*_ZnO_ when X-rays of various energy interact first with one of the ZnO NPs. In these simulations, 215 ZnO NPs were randomly placed on the surface of the SiO_2_ NP. In the inset, a magnification of the *E*_dep_ as a function of *r*_ZnO_ within the diameter of the ZnO NP (5 nm) is proposed.

The panels in Fig. 3 display the simulation results obtained with the code A for one SiO_2_/ZnO nanosystem. The conditions used for this code are described in Methods, Section 2.3, and depicted in Fig. 3a where a single SiO_2_ sphere (in white) is surrounded by ZnO NPs (in red). By means of code A, we investigated the impact of an increasing number of ZnO NPs attached to the silica surface (0, 10, 100 and 215 NPs) on the energy deposition process when 1 × 10^8^ X-ray photons, of the selected energy, are irradiating the system. Fig. 3b and Table 1 report the overall fraction of energy being deposited in each medium in the case of 215 ZnO NPs, regardless of where the primary interaction occurred. Results depict that, for 3.3 and 6 keV X-rays, most of the energy deposition occurs in SiO_2_. However, for 10 keV X-rays, thanks to ZnO's higher density and *Z*_eff_ value, most (about 75%) of the deposited energy is released in ZnO NPs despite their limited total volume.

**Table 1:** Percentage of E deposited in SiO_2_, ZnO, and air for each energy of X-ray beam, regardless of where the primary interaction occurs, according to the results from Code A, where 215 ZnO NPs (5 nm diameter) are randomly placed on the surface of a SiO_2_ NP (80 nm diameter)

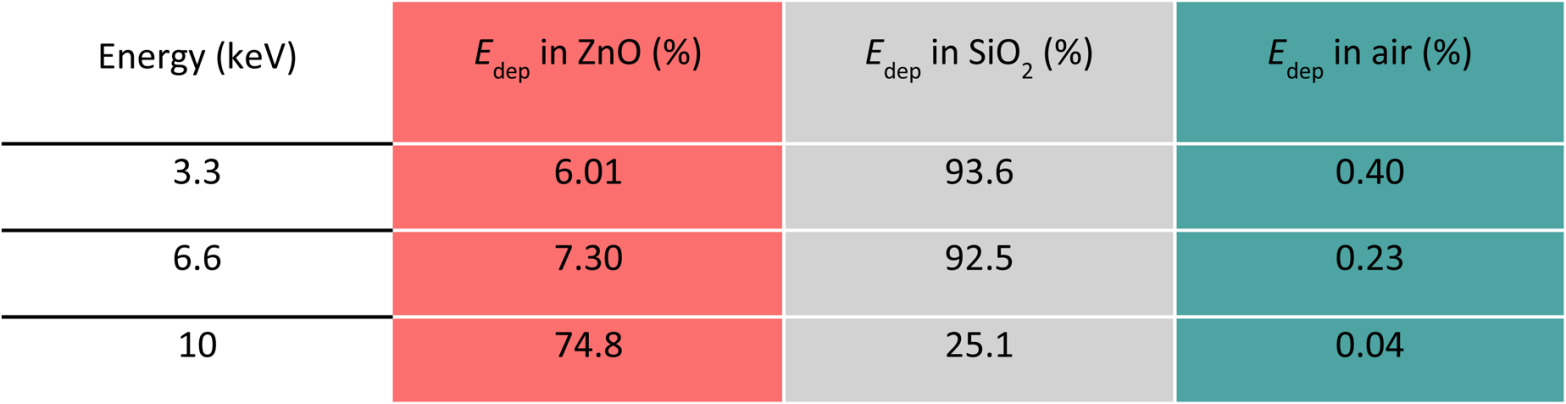

The *E*_dep_ profile *vs. r*_SiO_2__ rises as the volume of the respective spherical shell increases and then it suddenly drops at 40 nm, irrespective of the energy of the beam, when the edge of the SiO_2_ NP is reached. The residual amount of *E*_dep_, between 40 nm and 45 nm, can be ascribed to the interaction of the secondary electrons with ZnO NPs. Thus, when the primary interaction occurs in silica, only a small fraction of the energy is deposited outside the silica sphere. However, the fraction of the total *E*_dep_ in SiO_2_, regardless of where the primary interaction occurred, goes down while the beam energy rises, which is particularly obvious for 10 keV (Fig. 3b and Table 1).

Fig. 3f–h depict the profile of the deposited energy when the first X-ray interaction event occurs in one of the ZnO NPs. Here, the profile of deposited energy is expressed as a function of the distance, *r*_ZnO_, from the center of the ZnO NP where the primary interaction occurred, and it, again, increases with *r*_ZnO_ until the radius of the NPs is reached, *i.e.* 2.5 nm. As the energy of the incoming photons increases, the fraction of the deposited energy within the same ZnO NP where the primary interaction occurred grows, with a maximum reached for a 10 keV beam. In parallel, the energy *E*_dep_ deposited outside the ZnO particle after the interaction of the ionizing radiation with ZnO follows different paths, depending on the energy of the beam. Indeed, for X-ray energy of 3.3 keV and 6.6 keV, a fraction of the generated secondary electrons has enough kinetic energy to escape from the ZnO. The amount of kinetic energy of these carriers, and thus, energy released outside the NPs is expected to be larger for excitation with an X-ray beam at 6.6 keV with respect to the case beam at 3.3. keV. In fact, we can observe that the radial distribution of the deposited energy is spread over a broader distance range when 6.6 keV is considered. On the contrary, the *E*_dep_ outside the ZnO goes to zero as the X-ray beam at 10 keV is mostly absorbed by ZnO, due to the resonance with the absorption edge of Kα transition of zinc that is peaked at about 9.7 keV.^58^ Thus, the secondary charges do not possess the sufficient energy to steam out the NPs as their residual kinetic energy is relatively low (Fig. 3b and Table 1). The importance of the presence of dense ZnO is further supported by the results of code A for simulations where 0, 10, 100, and 215 ZnO NPs cover the silica sphere surface. Indeed, despite the diminution of the number of X-rays interacting with the SiO_2_/ZnO at 10 keV (Fig. S6 and Table S5†), the role of ZnO in harvesting the incoming photons with high energy dominates already for 100 ZnO NPs (Fig. S7 and Table S6†).

**Fig. 4:**
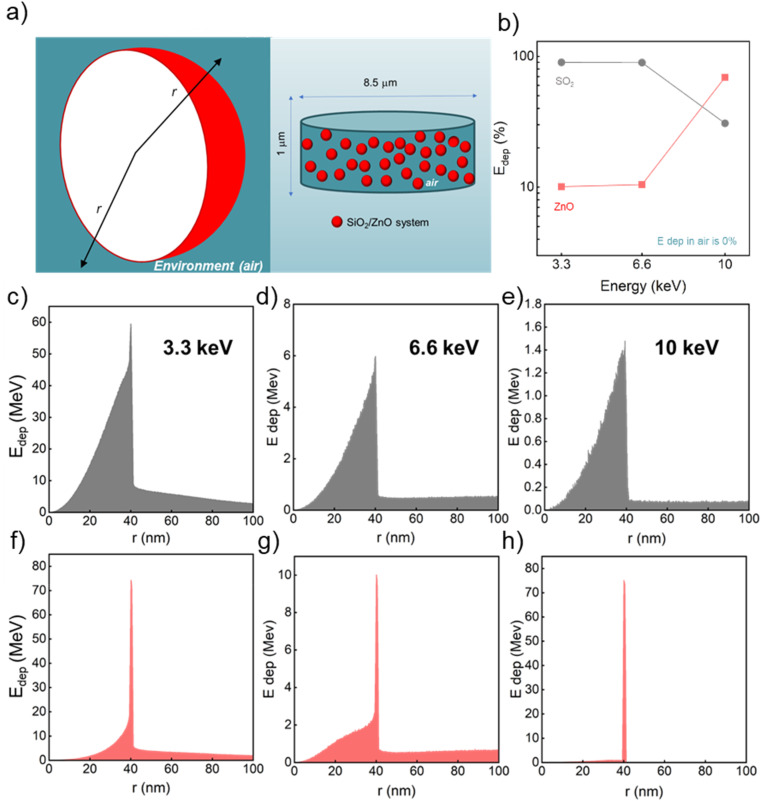
(a) Approximation of the SiO_2_/ZnO geometry is displayed where each SiO_2_ NP (white) is covered with a layer of 0.7 nm of ZnO (red). The thickness of the ZnO layer is calculated to provide a volume equivalent to 215 zinc oxide NPs (5 nm diameter), similarly to code A. A number of 5 × 10^4^ SiO_2_/ZnO (red spheres) are randomly placed in a cylinder (8.25 μm diameter, 1 μm height) filled with air (teal green). The geometry for code B simulates the experimental RL setup conditions. (b) Percentage of energy deposited (*E*_dep_ (%)) in ZnO and SiO_2_, from the data reported in Table 2 simulated using code B. (c–h) Energy deposited in the nanosystems plotted as a function of *r*, the distance to the center of the NP where the energy is deposited. *E*_dep_ is calculated for the three X-ray energies when the X-rays first interact with SiO_2_ (c–e) or ZnO (f–h).

To simulate the interaction of X-rays with a large number of NSs, the second code (code B) was developed, simulating a sample holder filled with ∼10^4^ silica spheres covered by a ZnO layer, whose volume equals the volume of 215 ZnO NPs of code A (Fig. 4a). As explained in the Method section, the replacement of the 215 ZnO NPs with a spherical shell of the same total volume was necessary to accommodate the computational capabilities. Fig. 4b and Table 2 report how the deposited energy is distributed among the different media regardless of where the primary interaction occurred. The dependence on the energy of the incoming photons is analogous to the one observed for code A (Fig. 3b and Table 1) with the exception of the energy deposited in air. Because code B considers a large number of packed NSs, only a limited volume is occupied by air. Consequently, the fraction of energy being deposited in air is negligible. In Fig. 4c–h, for the three X-rays energies considered, the spatial profile of the deposited energy in the SiO_2_/ZnO is reported as a function of *r*, where *r* is the distance between the point where the energy is being deposited and the centre of the SiO_2_ sphere. The profile of *E*_dep_ is calculated when the first interaction with the ionizing radiation occurs either in SiO_2_ (Fig. 4c–e) or in ZnO (Fig. 4f–h). In accordance with code A, the number of interacting photons over the 1 × 10^8^ incoming ones decreases when the X-ray energy increases (Fig. S8 and Table S7†). When the primary interaction occurs within a silica sphere, for energy of 3.3 keV, part of the energy is deposited within the same sphere. However, a significant fraction of energy is deposited outside the sphere (*r* > 40 nm), either in the surrounding medium or in other nearby SiO_2_/ZnO NSs. The narrow peak in the distribution, in proximity of *r* = 40 nm is related to the energy being deposited within the ZnO shell surrounding the SiO_2_ sphere. When the primary interaction occurs in the ZnO spherical shell, a significant fraction of energy is deposited at *r* = 40 nm for all beam energies, indicating that most of the secondary electrons do not escape the ZnO shell. Interestingly, in agreement with code A, the amount of energy deposited outside ZnO changes with the energy of the X-rays. With respect to the case of 3.3 keV, when 6.6 keV radiation is considered, a higher fraction of energy is deposited outside the ZnO layer because the higher kinetic energy of the secondary electrons allows them to travel longer distances. In the case of 10 keV, however, the photoelectric interaction with Ka electrons becomes possible. This turns out in the emission of low kinetic energy electrons that hardly exit the ZnO layer.

**Table 2:** Percentage of E deposited in SiO_2_, ZnO, and air for each X-ray beam energy according to the results from Code B. The deposited energy is distributed among the different media regardless of where the primary interaction occurred

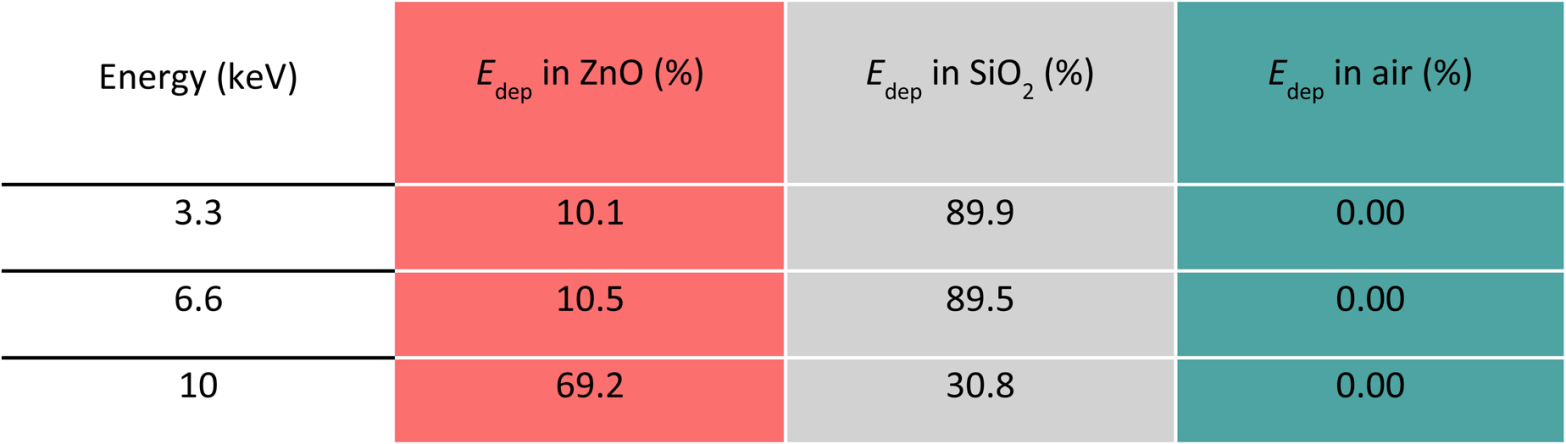

The results coming from codes A and B both point out the importance of the presence of dense ZnO around the medium-density silica sphere to favour the deposition of energy from the incoming X-ray beam with energies up to 10 keV. This is particularly evident at 10 keV as the presence of ZnO increases the probability of interaction with the incoming X-ray photons (Fig. S6 and S8†). The spatial energy deposition profiles reveal that, for the X-ray energy range considered, a relevant amount of the energy deposition occurs in the volume where the primary interaction occurs: due to their higher density with respect to the environment, both SiO_2_ and ZnO can be involved in stopping the ionizing radiation for each energy. However, for 3.3 keV energy, silica plays a dominant role in the interaction of the ionizing radiation; in turn, the presence of ZnO has a particularly positive effect on incoming X-rays of 10 keV. Moreover, at any energy, a limited amount of energy is deposited in the surrounding medium (air) outside the considered NS. In general, the probability of the interaction between matter and ionizing electromagnetic radiation, which is driven by the photoelectric effect, Compton scattering, and pairs production and expressed by the physical parameter of mass attenuation coefficient, is ruled by the density and *Z*_eff_ of the irradiated matter and the energy of the incoming ionizing radiation.^59,60^ At the energy of the incident X-ray beam of 3.3 keV, the photoelectric effect is the predominant type of interaction. Despite SiO_2_ featuring a lower density *Z*_eff_, with respect to ZnO, the deposition of energy in silica is favoured due to the bigger volume of SiO_2_ (26 800 nm^3^) compared to the one occupied by 215 NPs of ZnO (14 060 nm^3^). When the X-ray energy increases, the higher *Z*_eff_ value of ZnO becomes more important in enhancing the interaction with the incoming radiation. This is particularly true for 10 keV X-ray photons because of the X-ray absorption edge for the Kα transition of zinc.^58^ Consequently, 10 keV X-rays are absorbed by ZnO NPs, leading to the ionization of zinc core shells and to the generation of low-energy photo and Auger electrons that can further deposit their energy inside the ZnO NPs. However, the energy deposited is negligible outside ZnO for 10 keV photons (Fig. 3h and 4h) since, above the K-edge, the created charges have low energy impeding them from escaping from the ZnO itself. Upon exposure to 6.6 keV X-rays, the interaction and energy deposition mechanisms of either SiO_2_ or ZnO are not as efficient. This can be explained considering that, at this photonic energy, the energy deposition due to the presence of SiO_2_ starts to diminish, while the absorption of the energy by ZnO remains weak since the X-ray energy is below the zinc absorption K-edge.

Once the results from the numerical simulations are assessed, the TCPP emission sensitization would be expected to follow the same energy-dependent behaviour foreseen by code B. To try to evidence the Zn contribution to the X-ray interaction, we performed RL measurements as a function of the accelerating voltage on two samples (Fig. S9†). One consists of ZnO/SiO_2_ @APTES@Porp 1%, the second one is identical but without ZnO NPs. Unfortunately, the comparison between the experimental data and the numerical simulations appears to be not straightforward. The most problematic point in this comparison is related to the fact that our X-ray source is not monochromatic as is the case for the Monte Carlo modelling. In fact, given the acceleration voltage (up to 32 kV) and the tungsten target, most of the emitted X-rays are being generated by bremsstrahlung resulting in a continuous energy distribution over imposed on a few monochromatic tungsten lines. Only the average of such a distribution approximates the one used for the numerical simulations. This results in a smoothing of all the phenomena predicted by the simulations, especially those occurring in ZnO. On one side, when moving from an average energy of 3.3 keV to 6.6 keV the reduction in the overall RL intensity, which would be expected from the decreased interaction probability with the ionizing radiation predicted by the code B (Fig. S11c†) is absent (ESI Fig. S11b†). However, it is worth noticing that, due to computational constraints, the modelled sample dimensions were downscaled by three orders of magnitude with respect to the real ones, resulting in a thickness of 1 μm rather than 1 mm. As a consequence, the simulation fails to account for the interactions occurring in most of the sample thickness progressively underestimating the total deposited energy while the acceleration voltage increases. On the other hand, due to the continuous distribution of X-ray energy in the RL experiment, the energy threshold due to the Zn Kα absorption line is not clearly detectable. In fact, at an acceleration voltage of 20 kV (corresponding to a mean energy of 6.6 keV) a significant fraction of photons has an energy exceeding 10 keV. Nevertheless, the normalized RL spectra on SiO_2_ @APTES@Porp 1% and ZnO/SiO_2_ @APTES@Porp 1%, shown in Fig. S10,† evidence the intensity dependence as a function of the tube voltage and a better performance of the ZnO containing sample, particularly for the highest X-rays energies. This result can be seen in agreement with Fig. S11b† where the maximum TCPP red luminescence sensitization occurs for the highest deposited energy amount for X-rays at an energy of 10 keV.

## 4 Conclusions

In this work, the radioluminescence emissions of hybrid NS composed of heavy SiO_2_/ZnO functionalized using porphyrin molecules were investigated upon X-ray irradiation at increasing energy in the range of keV (up to 10 keV). Using these nanoparticles for biomedical applications would require the use of X-rays with higher energies, as X-rays with energies below 10 keV would lack penetration in tissues and could thus not be used to deliver radiotherapy *in vitro* or *in vivo*. However, this study provides a fundamental understanding of energy partitioning in the nanosystem and allows to identify the specific role of each nanosystem's composing element. The spatial profile of the energy deposition occurring when X-rays interact with the SiO_2_/ZnO was simulated using the Monte Carlo Geant4 toolkit. Two models were developed, one to explore the role of an increasing number of ZnO NPs on the surface and the other one to represent the experimental conditions used during RL measurements. By combining the RL experiments with the computational studies, we propose the results suggesting an important role of ZnO in stopping the ionizing radiation for energy of tens of keV. Thus, our findings highlight the importance of detailed knowledge of how the energy deposited by ionizing radiation is distributed in the specific NS system employed. This insight, in fact, is crucial in determining the necessary strategies to improve the energy transfer towards TCPP and thus increase its luminescence. In fact, besides the radiative energy transfer activation that can partially excite the PS on the surface of SiO_2_/ZnO and induce its red luminescence, an efficient and improved energy deposition process occurring in the nanosystems, involving both silica and ZnO according to the energy of the X-rays, plays a beneficial role in the TCPP luminescence enhancement and hopefully in the activation of its cytotoxicity.

This discovery can help to guide the design of future hybrid NS to be used in clinics, where a specific augmentation of energy deposition mechanism – in the presence of X-ray beams with energy reaching hundreds of keV – is foreseen and boosted.

## Data availability

Data for this article, including raw data and editable graphs are available at https://doi.org/10.17632/bw9sj9mhmh.1, as well as they are part of the ESI.† The code for Geant4 can be found at https://geant4.web.cern.ch/. The version of the code employed for this study is geant4-10-06-patch-01 version. Outputs of the code are available at https://doi.org/10.17632/bw9sj9mhmh.1. The data supporting this article are included as part of the ESI:† (1) TGA analyses. (2) RL spectral deconvolution using the Levenberge–Marquardt algorithm. (3) Monte Carlo simulations results using the GEANT4 toolkit (version 4.10.6 patch 01). Data for this article, including raw data and editable graphs of: Gaussian spectral reconstruction – fit results; MONTECARLO Geant4 Simulation outputs (Code A and Code B); Energy deposition *vs.* distance are available at http://board.unimib.it at Villa, Irene; Fasoli, Mauro (2024), “The role of energy deposition on the luminescence sensitization in porphyrin-functionalized SiO2/ZnO nanoparticles under X-rays excitation”, Bicocca Open Archive Research Data, V1, https://doi.org/10.17632/bw9sj9mhmh.1. The code for Geant4 can be found at https://geant4.web.cern.ch/. The version of the code employed for this study is geant4-10-06-patch-01.

## Author contributions

Conceptualization, I. V., M. F., A. V., and R. C.; spectroscopic and morphological analysis, R. C. and S. M.; investigation, I. V., R. C., A. L. B., M. F., and S. M.; supervision, I. V., S. M., A. L. B., M. F., M. D. A., B. D. C., R. S., and A. V.; validation, I. V., S. M., A. L. B., M. F., M. D. A., B. D. C., R. S., and A. V.; visualization, R. C., S. M., I. V., and M. F.; writing – original draft, R. C., I. V., and S. M.; writing – review and editing, I. V., A. L. B., and M. F. All authors have read and agreed to the published version of the manuscript.

## Conflicts of interest

The authors declare no conflict of interest.

## Supplementary Material

NA-OLF-D4NA00640B-s001
