# Supplementary material for: The role of energy deposition on the luminescence sensitization in porphyrin-functionalized SiO2/ZnO nanoparticles under X-ray excitation
Source: Nanoscale Adv. 2025 Jan 8;7(5):1464–74. doi: 10.1039/d4na00640b (PMC11758577; doi:10.1039/d4na00640b)
Supplement: NA-007-D4NA00640B-s001 [file NA-007-D4NA00640B-s001.pdf]

Supplementary Information (SI) for Nanoscale Advances.
This journal is © The Royal Society of Chemistry 2025

Supporting Information for

The role of energy deposition on the luminescence sensitization in porphyrin functionalized SiO_2/ZnO nanoparticles under X-rays excitation

Irene Villa [1], Roberta Crapanzano [1], Silvia Mostoni [1,2], Anne-Laure Bulin [3], Massimiliano D'Arienzo [1,2], Barbara Di Credico [1,2], Anna Vedda [1], Roberto Scotti [1,2,4], and Mauro Fasoli [1]*

[1] Department of Materials Science, University of Milano - Bicocca, Via Cozzi 55, I-20125, Milano, Italy

[2] INSTM, University of Milano - Bicocca, Via Cozzi 55, I-20125, Milano, Italy

[3] University Grenoble Alpes, Inserm U1209, CNRS UMR5309, Cancer Targets and Experimental Therapeutics Team, Institute for Advanced Biosciences, 38000 Grenoble, France

[4] Institute for Photonics and Nanotechnologies-CNR, Via alla Cascata 56/C, 38123 Povo (TN), Italy

*Correspondence: irene.villa@unimib.it

TGA analysis

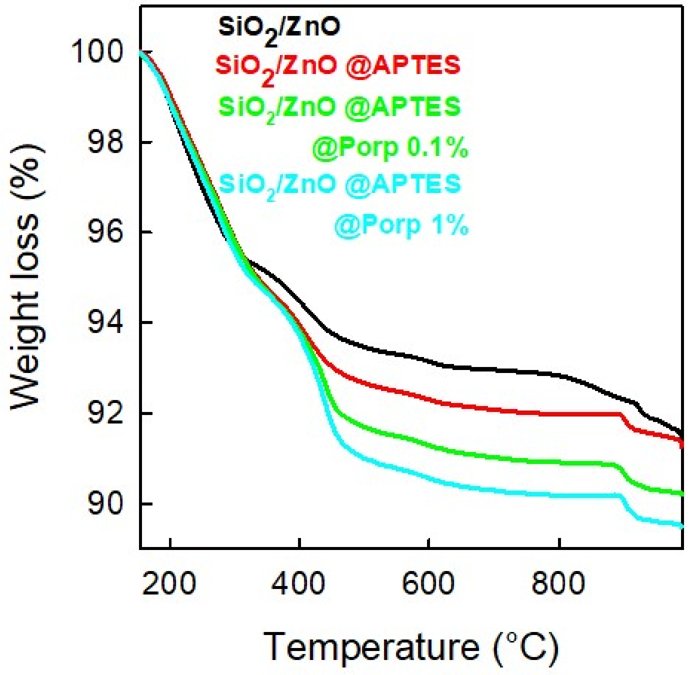

Figure S1: TGA profiles of ZnO/SiO_2 based materials in the temperature range 150-1000 °C.

Sample	TGA results			
	$\Delta W_{150-1000\ °C}$ (%)	TCPP amount (wt%)	Nominal TCPP (wt%)	Ratio n molecules TCPP/APTES
SiO_2/ZnO	8.5	-	-	-

SiO_2/ZnO@APTES	8.7	-	-	-
SiO_2/ZnO@APTES@Porp 0.1%	9.5	0.8	0.3	0.3
SiO_2/ZnO@APTES@Porp 1%	10.8	2.1	2.7	0.8

Table S1**:** Amounts of APTES and TCPP anchored over SiO_2 calculated from TGA results (according to the method reported in [38]). The uncertainty on the quantitative determination was evaluated as a mean of three different TGA analysis run on each sample.

UV-Vis absorption measurements

TCPP amount in SiO_2/ZnO@APTES@Porp 0.1% and SiO_2/ZnO@APTES@Porp 1% was assessed through UV-Vis experiments by measuring the TCCP absorbance of highly diluted powder dispersions (1.3 mg powder/mL in DMF, corresponding to 10^{-3} M of ZnO).

The linear increase of the absorbance at 520 nm as a function of the TCPP concentration was previously verified by measuring the absorbance of TCPP solutions at known concentration (in the range: 10^{-6} – 7×10^{-5} M). By doing this, a calibration curve was obtained by using the Lambert Beer equation and the molar extinction coefficient at 520 nm was calculated as reported in 38.

The data are summarized in Table S2.

Sample	Abs	C_{TCPP} ($A_{520\ nm}$) [M]	TCPP amount (wt%)
SiO_2/ZnO@APTES@Porp 0.1%	0.005±0.002	$6.1*10^{-6}$	0.37
SiO_2/ZnO@APTES@Porp 1%	0.032±0.002	$3.6*10^{-5}$	2.21

Table S2. Absorbance (Abs) values of Q band at 520 nm and calculated TCPP amounts of SiO_2/ZnO@APTES@Porp samples. Data were elaborated by using the Lambert-Beer equation, focusing on the absorbance value of the first TCPP Q-band at 520 nm (molar extinction coefficient at 520 nm $\varepsilon_{\_520}$ = 8900 M^{-1} cm^{-1}, optical path length d = 0.1 cm), as in [38].

Gaussian spectral reconstruction.

All the RL spectra were recorded at room temperature and corrected for the spectral response of the detector. The RL spectra were fitted as the sum of the same Gaussian components (Figures S2-S4), whose complete sets of used parameters - energy position (E, eV), full width at high maximum (FHWM, eV), and integral (arb. units) - are reported in Tables S3 and S4. To perform the fit procedure, we 1) used the minimum number of bands required for a satisfactory reproduction of the experimental curves, 2) considered the E bands values reported in the literature for ZnO and porphyrin emissions as well as for porphyrins' Q bands, and 3) E and full width at half maximum (FWHM) values were free to vary of ± 0.02 eV.

	**E (eV)**	**FWHM (eV)**
ZnO	2.22	0.77
	3.09	0.6

**TCPP**	1.55	0.32
	1.69	0.09
	1.8	0.08
	1.96	0.11
**Q Bands**	2.01	0.1
	2.2	0.1
	2.34	0.1
	2.5	0.1

Table S3: Fit parameters obtained from the Gaussian deconvolution of the RL spectra recorded under three diverse mean energies of the X-ray beam (3.3 keV, 6.6 keV, 10 keV) in the NS.

	**X-rays mean energy 3.3 keV**				
	**Integrals (arbitrary units)**				
**E bands (eV)**	**SiO_2/ZnO**	**SiO_2/ZnO APTES**	**SiO_2/ZnO APTES TCPP 0.1%**	**SiO_2/ZnO APTES TCPP 1%**	**TCPP**
**1.55**			0.013	0.02	0.020
**1.69**			0.007	0.009	0.008
**1.8**			0.008	0.006	6.9 E-04
**1.96**			0.011	0.004	
**2.22**	0.17	0.18	0.063	0.016	
**2.01**			-0.003	-0.001	
**2.2**			-0.004	-8.3 E-04	
**2.34**			-0.002	-8.8 E-04	
**2.5**			0	-6.5 E-05	
**3.09**	0.011	0.008			
	**X-rays mean energy 6.6 keV**				
	**Integrals (arbitrary units)**				
**E bands (eV)**	**SiO_2/ZnO**	**SiO_2/ZnO APTES**	**SiO_2/ZnO APTES TCPP 0.1%**	**SiO_2/ZnO APTES TCPP 1%**	**TCPP**
**1.55**			0.078	0.13	0.071
**1.69**			0.052	0.06	0.031
**1.8**			0.058	0.038	0.003
**1.96**			0.064	0.018	
**2.22**	1.33	1.11	0.38	0.093	
**2.01**			-0.008	-2.2 E-04	
**2.2**			-0.022	-0.006	
**2.34**			-0.015	-0.005	

**2.5**			-5.6 E -04	-0.001	
**3.09**	0.072	0.046			
	**X-rays mean energy 10 keV**				
	**Integrals (arbitrary units)**				
**E bands (eV)**	**SiO_2/ZnO**	**SiO_2/ZnO APTES**	**SiO_2/ZnO APTES TCPP 0.1%**	**SiO_2/ZnO APTES TCPP 1%**	**TCPP**
**1.55**			0.13	0.20	0.10
**1.69**			0.087	0.098	0.046
**1.8**			0.10	0.063	0.006
**1.96**			0.11	0.034	
**2.22**	2.31	1.82	0.62	0.14	
**2.01**			-0.01	-0.004	
**2.2**			-0.034	-0.008	
**2.34**			-0.023	-0.008	
**2.5**			0	-0.001	
**3.09**	0.12	0.077			

Table S4: Integrals of the Gaussian components of the RL spectra recorded under excitation with X-Ray photon of 3.3 / 6.6 / 10 keV mean energy of the investigated nanosystems.

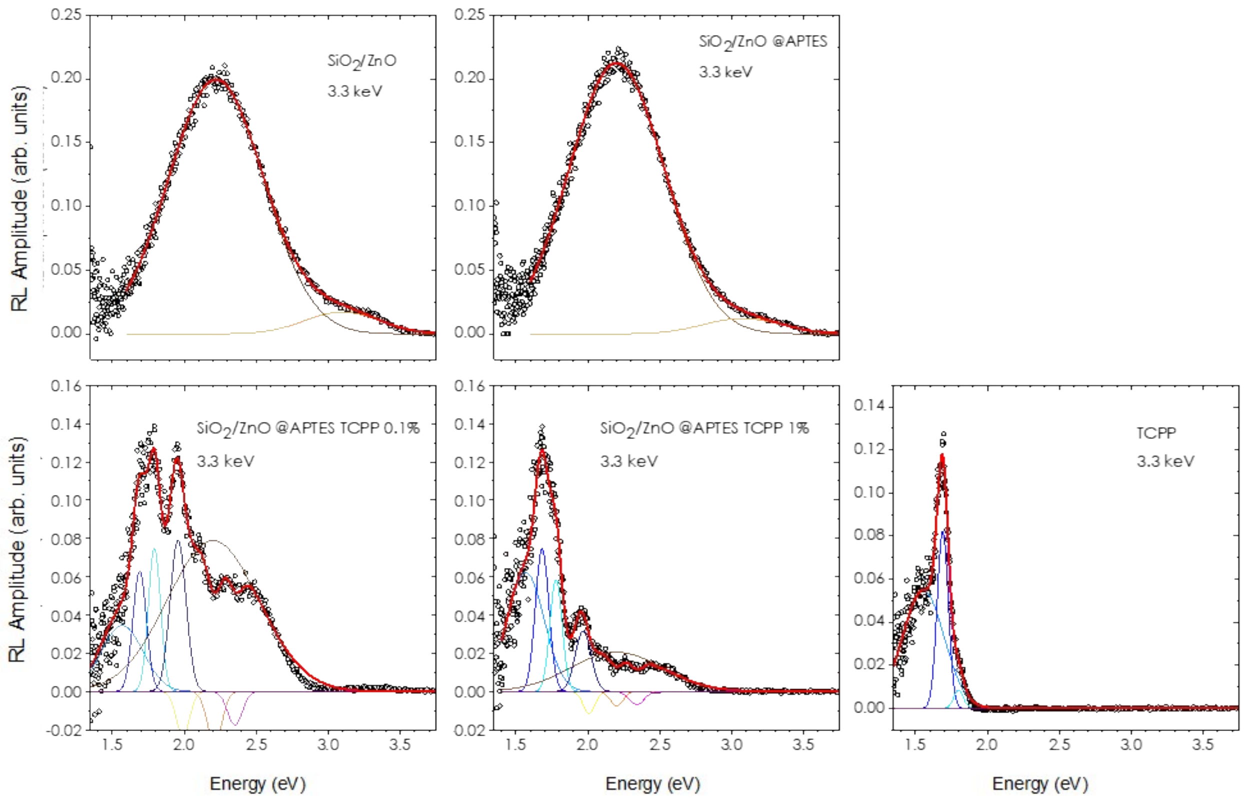

Figure S2: Gaussian deconvolution of RL spectra recorded under excitation with X-Ray photons of 3.3 keV mean energy of the bare, the APTES decorated, the TCPP functionalized SiO_2/ZnO nanosystems and the porphyrin. Gaussian components (solid lines) obtained by numerical fit are shown together with experimental curves (black empty circle lines). The curve representing the whole numerical fit (red line) is superimposed to the experimental data. Gaussian components representative of the absorption of the porphyrin in the typical Q-bands are displayed with negative area in correspondence of dips in the radioluminescence spectra. The sets of all parameters of the deconvolution are listed in Tables S3-S4.

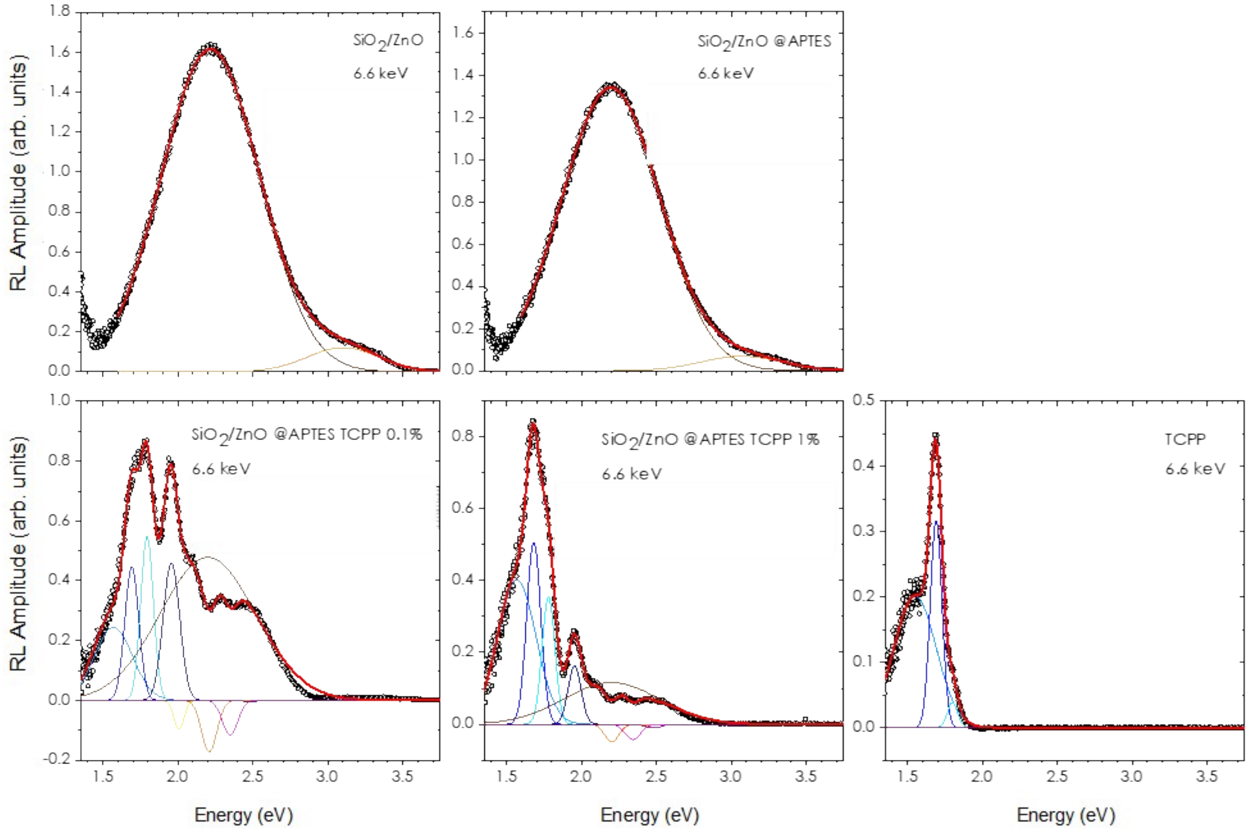

Figure S3: Gaussian deconvolution of RL spectra recorded under excitation with X-Ray photons of 6.6 keV mean energy of the bare, the APTES decorated, the TCPP functionalized SiO_2/ZnO nanosystems and the porphyrin. Gaussian components (solid lines) obtained by numerical fit are shown together with experimental curves (black empty circle lines). The curve representing the whole numerical fit (red line) is superimposed to the experimental data. Gaussian components representative of the absorption of the porphyrin in the typical Q-bands are displayed with negative area in correspondence of dips in the radioluminescence spectra. The sets of all parameters of the deconvolution are listed in Tables S3-S4.

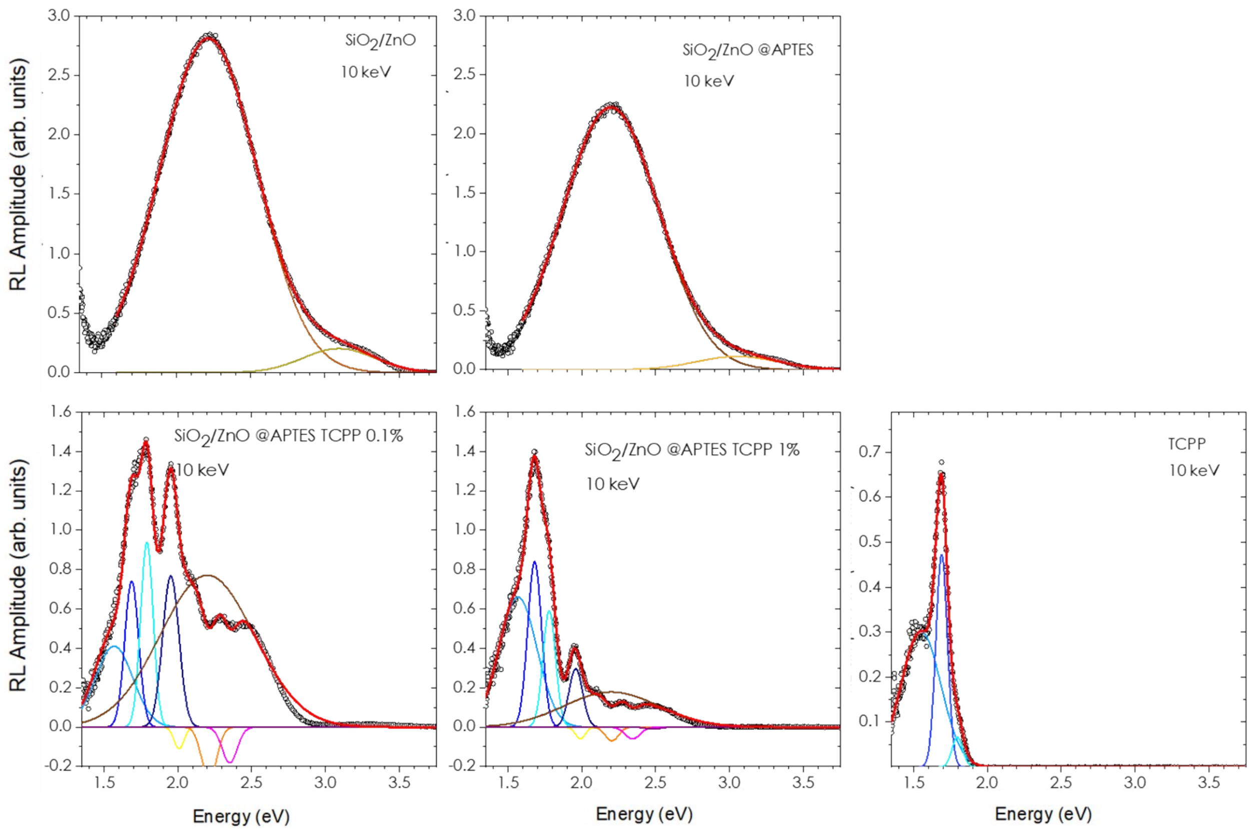

Figure S4: Gaussian deconvolution of RL spectra recorded under excitation with X-Ray photons of 10.0 keV mean energy of the bare, the APTES decorated, the TCPP functionalized SiO_2/ZnO nanosystems and the porphyrin. Gaussian components (solid lines) obtained by numerical fit are shown together with experimental curves (black empty circle lines). The curve representing the whole numerical fit (red line) is superimposed to the experimental data. Gaussian components representative of the absorption of the porphyrin in the typical Q-bands are displayed with negative area in correspondence of dips in the radioluminescence spectra. The sets of all parameters of the deconvolution are listed in Tables S3-S4.

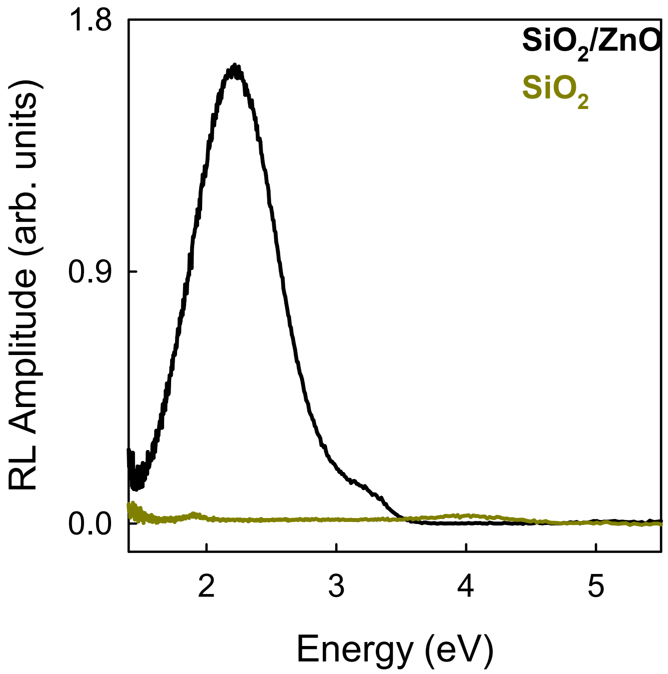

Figure S5: RL spectrum at 6.6 keV of the SiO_2 NPs compared to that of the SiO_2/ZnO NPs

Monte Carlo simulations

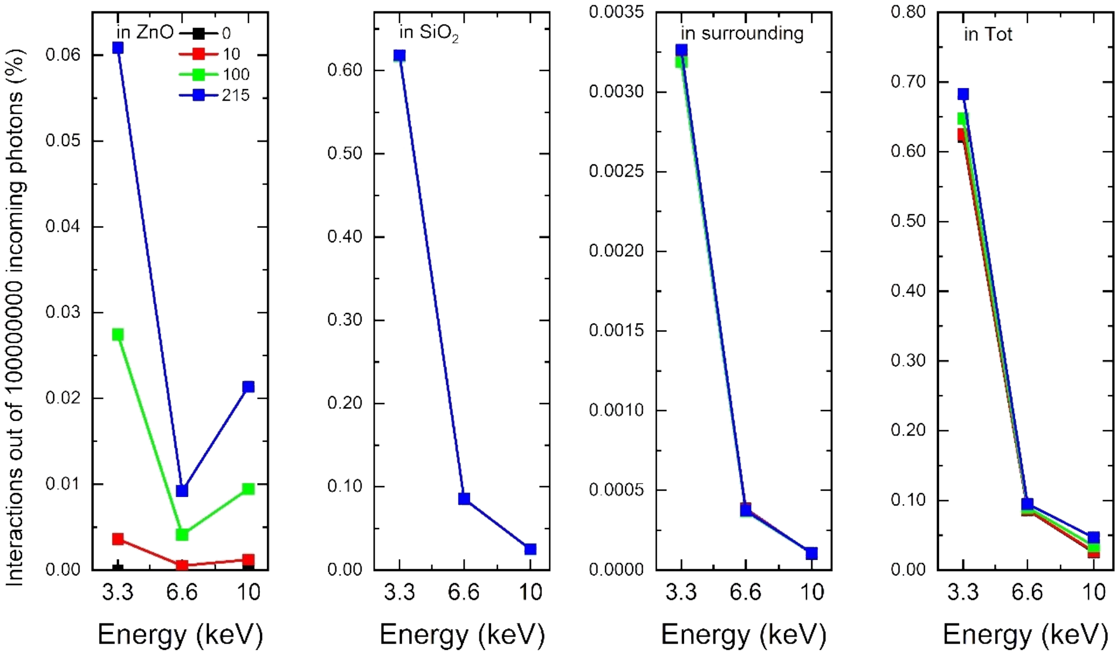

Figure S6: Percentage of interacting photons out of the incoming ones (1x10^8) within ZnO, SiO_2, and air, as a function of the energy of the X-ray beam. For the interaction of the photons in ZnO, the dependence of the interaction is displayed according to the amount of NPs on silica surface. Data were obtained by code A simulation and reported in Table S5.

Percentage of incoming photons interacting in the various component of the nanosystem					
**X-ray energy (keV)**	**Number of ZnO NPs**	%ZnO	$\%SiO_2$	%air	**% tot**
**3.3**	**0**	0	6.18E-01	3.21E-03	**6.21E-01**
**6.6**		0	8.58E-02	3.85E-04	**8.61E-02**
**10**		0	2.52E-02	1.02E-04	**2.53E-02**
**3.3**	**10**	3.60E-03	6.18E-01	3.21E-03	**6.25E-01**
**6.6**		5.01E-04	8.57E-02	3.83E-04	**8.66E-02**
**10**		1.21E-03	2.51E-02	1.04E-04	**2.64E-02**
**3.3**	**100**	2.75E-02	6.17E-01	3.19E-03	**6.48E-01**
**6.6**		4.13E-03	8.55E-02	3.68E-04	**9.00E-02**
**10**		9.45E-03	2.51E-02	1.06E-04	**3.46E-02**
**3.3**	**215**	6.09E-02	6.18E-01	3.27E-03	**6.83E-01**
**6.6**		9.20E-03	8.53E-02	3.74E-04	**9.49E-02**
**10**		2.14E-02	2.51E-02	1.07E-04	**4.66E-02**

Table S5: Percentage of X-rays photons that interact with ZnO, SiO_2, and air over the $1x10^8$ incoming photons simulated in code A. Results are presented for 0, 10, 100, and 215 ZnO NPs and for X-rays energies of 3.3 keV, 6.6 keV, and 10 keV.

E_{dep} (%)				
**X-ray energy (keV)**	**Number of ZnO nanoparticles**	**in ZnO**	**in SiO_2**	**in air**
3.3	**0**	0.00	99.60	0.40
6.6		0.00	99.78	0.22
10		0.00	99.85	0.15
3.3	**10**	0.35	99.25	0.40
6.6		0.39	99.38	0.22
10		14.75	85.12	0.13
3.3	**100**	2.80	96.80	0.40
6.6		3.43	96.34	0.23

10		57.16	42.77	0.06
3.3	**215**	6.01	93.59	0.40
6.6		7.30	92.47	0.23
10		74.83	25.14	0.04

Table S6: Percentage of energy deposited in ZnO, SiO_2, and air resulted from code A for 0, 10, 100, and 215 zinc oxide NPs and for X-rays energies of 3.3 keV, 6.6 keV, and 10 keV.

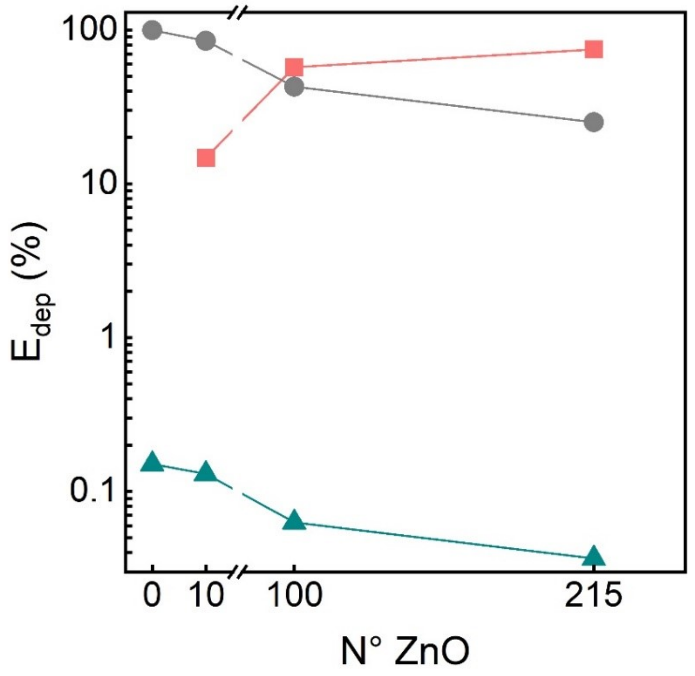

Figure S7: Fraction on E_{dep} (%) in NS vs number of ZnO at 10 keV in code A, according to the data in Table S6.

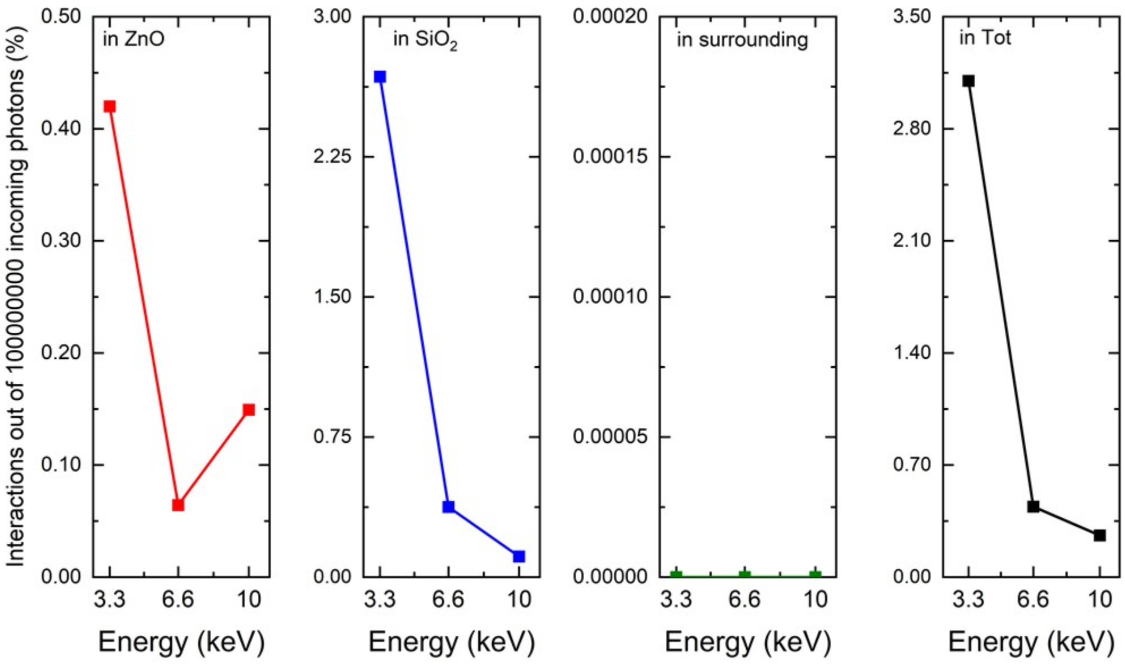

Figure S8: Percentage of interacting photons out of the incoming ones ($1x10^8$) within ZnO, SiO_2, and air, as a function of the energy of the X-ray beam obtained with code B. The displayed data are reported in Table S7.

**Percentage of incoming photons interacting in the various component of the nanosystem**				
**X-ray energy (keV)**	**%ZnO**	**%SiO_2**	**%air**	**%Total**
**3.3**	0.42	2.68	0.00	**3.10**
**6.6**	0.06	0.38	0.00	**0.44**
**10**	0.15	0.11	0.00	**0.26**

Table S7: Percentage of the incoming X-rays photons that interact with ZnO, SiO_2, and air over the $1x10^8$ incoming photons in code B. Results are presented for X-rays energies of 3.3 keV, 6.6 keV, and 10 keV. The number of X-rays photons interacting in air is null due to the large amount of NS ($5x10^4$) filling the cylindric volume representing the experimental sample holder and, thus, to the limited volume occupied by air.

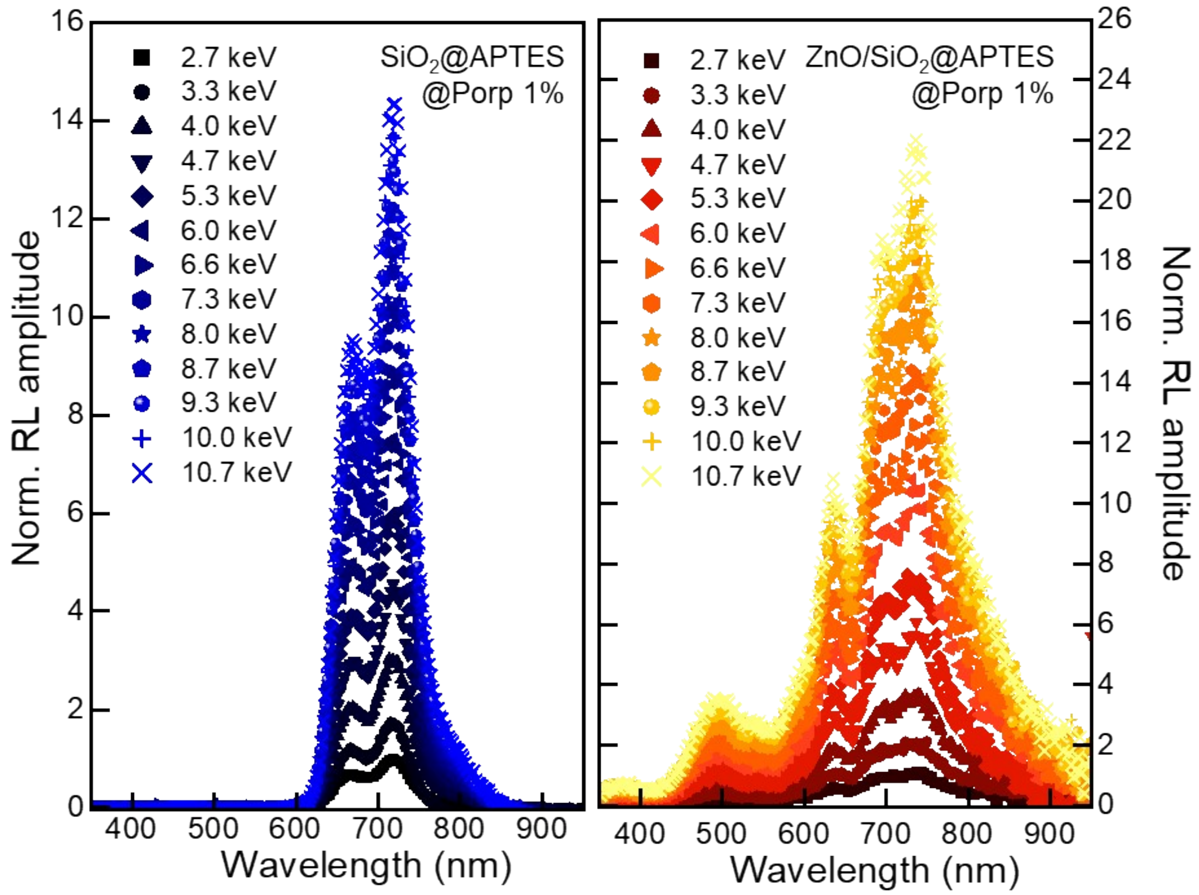

Figure S9: RL data recorded on SiO_2 NPs functionalized by TCPP, SiO_2@Porp 1%, (left) and on SiO_2/ZnO@APTES@Porp 1% system (right) under soft X-.rays beam with mean energies from 2.7 keV to 10.7 keV. The sensitization effect on TCPP RL emission in presence of ZnO was evidenced by normalizing the RL spectra to the RL acquired for the lowest excitation energy at 2.7 keV.

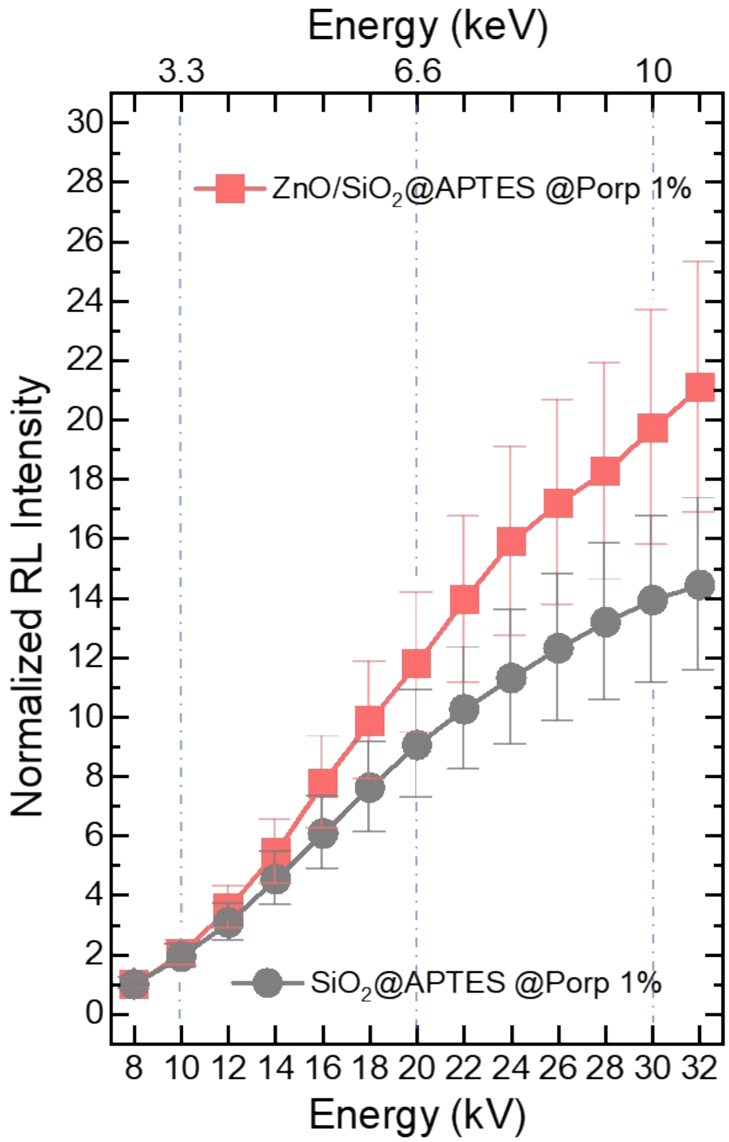

Figure S10: RL sensitization of TCPP red emission as a function of the incoming X-rays photons energy in a sample of SiO_2 functionalized by 1 wt% of TCPP (SiO_2@APTES@Porp 1%) and in SiO_2/ZnO functionalized with the same TCPP content (SiO_2/ZnO@APTES@Porp 1%). RL intensities of the samples were calculated by integrating the typical porphyrin emission bands over the red spectral range (600-800 nm) from the data displayed in Fig.S9. For clarity, the energy of the X-rays are expressed in terms of the X-ray tube voltage and of the mean X-rays energy estimated as 1/3 of the maximum voltage. The error bars refer to an experimental uncertainty of 20%.

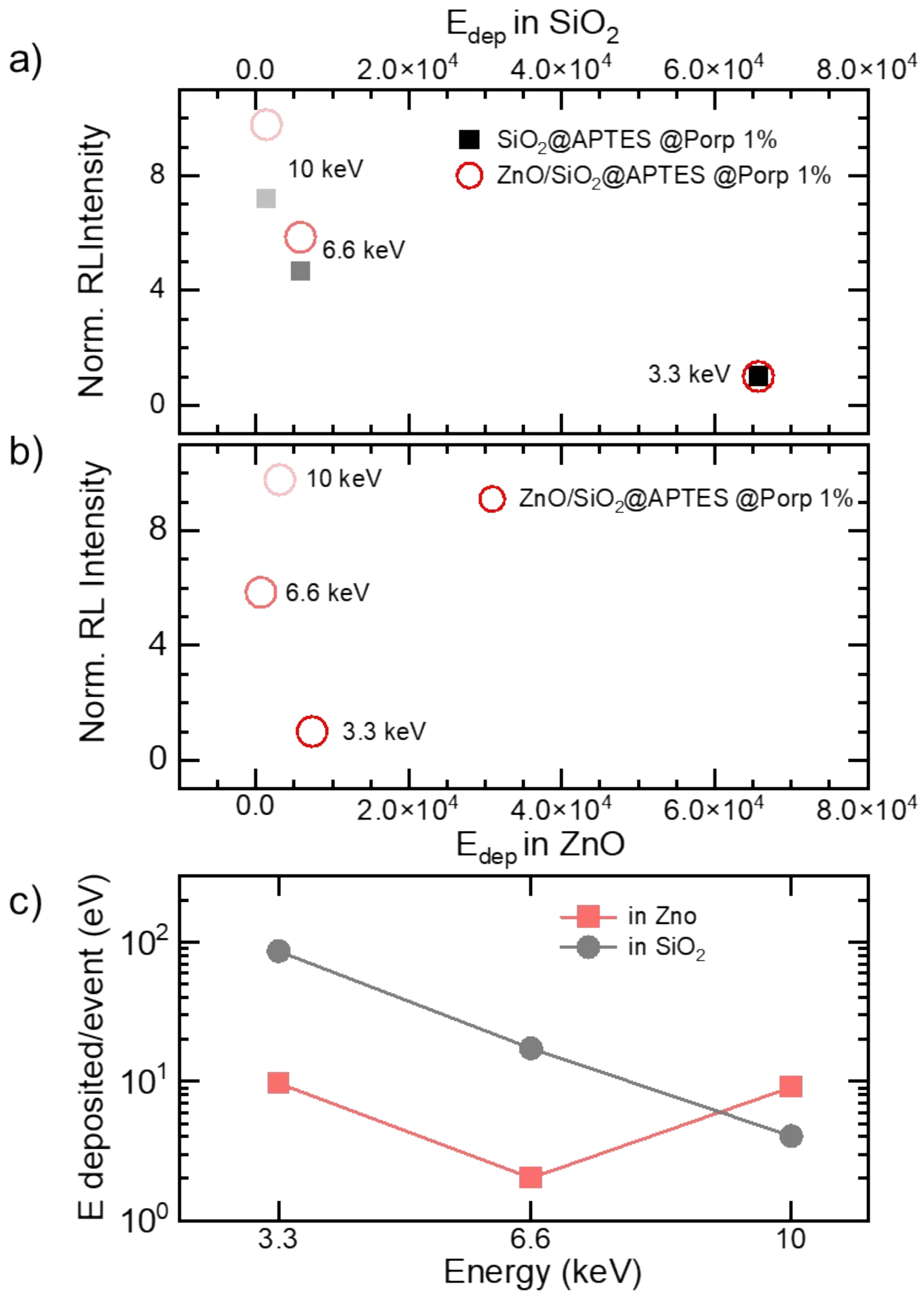

Figure S11: a) RL intensities of two measured samples of SiO_2@APTES @Porp 1% and ZnO/SiO_2@APTES @Porp 1% of 1 mm thickness as a function of the energy deposited in SiO_2 and b) RL intensities as a function of the energy deposited in ZnO for the same sample of ZnO/SiO_2@APTES @Porp 1%. The energies deposited in the two materials calculated from code B outcomes (reported in c)). The continuous energy distribution spectra of the X-rays tube used for RL experiments at 10 kV, 20 kV, and 30 kV operating voltage were simulated by XMI-MSIM tool designed for predicting the spectral response of energy-dispersive X-ray fluorescence spectrometers using Monte Carlo simulations. [1] X-rays mean energies were assumed to be 3.3 keV, 6.6 keV, and 10 keV; c) Energy deposited per event (i.e. per X-ray photon irradiating the sample regardless of where the primary interaction) as a function of the X-rays energy. Data were calculated by code B for a sample of 1 μm thickness and X-rays with monochromatic energies of 3.3 keV, 6.6 keV, and 10 keV

REFERENCE

1. Schoonjans, Tom, et al. "A general Monte Carlo simulation of energy dispersive X-ray fluorescence spectrometers—Part 5: Polarized radiation, stratified samples, cascade effects, M-lines." *Spectrochimica Acta Part B: Atomic Spectroscopy* 70 (2012): 10-23.
